# Recombinant adenoviruses application for cancer vaccines: from genetic design to clinical translation

**DOI:** 10.3389/fonc.2026.1824087

**Published:** 2026-07-13

**Authors:** Nada A. Almarghalani, Magdah A. Ganash, Mona G. Alharbi, Tareq Abualfaraj, Almohanad A. Alkayyal, Ahmad Bakur Mahmoud

**Affiliations:** 1 Department of Biological Sciences, Faculty of Science, King Abdulaziz University, Jeddah, Saudi Arabia; 2 Immunology Research Program, King Abdullah International Medical Research Center, Jeddah, Saudi Arabia; 3 Health and Life Research Center, Taibah University, Madinah, Saudi Arabia; 4 Department of Basic Medical Sciences, College of Medicine, Taibah University, Madinah, Saudi Arabia; 5 Laboratory Technology, Faculty of Applied Medical Sciences, University of Tabuk, Tabuk, Saudi Arabia; 6 Department of Clinical Laboratory Sciences, College of Applied Medical Sciences, Taibah University, Madinah, Saudi Arabia

**Keywords:** adenoviral vector, cancer immunotherapy, oncolytic adenovirus, replication-deficient adenovirus, tumor-selective oncolytic adenoviruses

## Abstract

Cancer remains a major global health challenge, with conventional therapies often limited by toxicity and insufficient efficacy. Oncolytic virotherapy has emerged as a promising immunotherapeutic approach, utilizing genetically engineered viruses to selectively target and destroy cancer cells while stimulating anti-tumor immune responses. Among various oncolytic viruses, adenoviruses (HAdVs) have been extensively studied due to their ability to be genetically modified for enhanced tumor selectivity and immunogenicity. This review explores the mechanisms by which adenovirus vectors function as cancer immunotherapies, detailing their genetic modifications to improve tumor targeting, replication, and immune activation. Additionally, we highlight key clinical trials that have evaluated adenovirus-based therapies in different cancer types, emphasizing their efficacy, safety, and potential for future therapeutic applications.

## 1 Introduction

Cancer remains a leading cause of morbidity and mortality worldwide, characterized by the uncontrolled proliferation of malignant cells (1). Epidemiological data indicate that approximately 2 million new cases and over 600,000 cancer-related deaths are reported annually in the United States, with global incidence continuing to rise (2, 3). Despite advances in early detection and conventional therapies, many cancers remain difficult to treat, often resulting in poor prognosis (4).

Conventional cancer treatments, including chemotherapy and radiotherapy, frequently exhibit limited efficacy and are associated with significant toxicities, which can compromise patient outcomes and quality of life (5). These limitations have motivated the development of novel therapeutic strategies that are both effective and well-tolerated. Among these, cancer immunotherapy has emerged as a promising approach, aiming to harness the patient’s immune system to specifically recognize and eliminate malignant cells. Immunotherapeutic modalities include immune checkpoint inhibitors, chimeric antigen receptor (CAR) T cells, cytokine therapies, and oncolytic viruses (6).

Oncolytic viral (OV) therapies are engineered viruses designed to selectively infect and destroy cancer cells while stimulating antitumor immune responses. Since the FDA approval of the first OV therapy, talimogene laherparepvec (T-VEC), in 2015, research into OVs has expanded significantly (7). Among OVs, human adenoviruses (HAdVs) have received considerable attention due to their ability to be genetically modified, their high replication efficiency in tumor cells, and their capacity to stimulate antitumor immunity (8).

HAdV-based vectors are generally classified into two groups: (1) replication-deficient adenoviruses, which are incapable of self-replication and are primarily used as gene therapy vectors, and (2) conditionally replicating, oncolytic adenoviruses (OAds), which selectively replicate in and destroy cancer cells (9). Beyond direct tumor lysis, OAds release tumor-associated antigens that trigger inflammation, converting immunologically “cold” tumors into “hot” tumors and enhancing immune-mediated clearance (10). This review provides a comprehensive overview of recent advances in OAd development and highlights strategies aimed at improving tumor selectivity, safety, and clinical efficacy.

## 2 Immunotherapy strategies targeting tumor microenvironment

The tumor microenvironment (TME) consists of tumor cells, fibroblasts, and immune cells associated with cancer cells, as well as the circulatory and blood vessels of the tumor (11). Tumor cells generally display uncontrolled cellular proliferation and rapid spread, causing damage and impairment in adjacent normal cells. To understand tumor immunity and develop immunotherapy for cancer, it is crucial to understand the characteristics of tumor antigens and the mechanism of the immune system response against malignancies. These characteristics include tumor antigens that stimulate targeted adaptive immune responses, that prevent and suppress the growth and spread of cancer (12). The TME exhibits the phenotypic traits of cancer cells, which shield them from the host immune system and contribute significantly to their invasion and migration (13). Significantly, in recent years scientists have examined the correlation between TME and the formation of tumors, which has yielded a highly promising area of study in cancer research (14).

One of the crucial hallmarks of tumors is the long-term state of chronic inflammation, which contributes to the formation of tumors by stimulating their proliferation, survival, and metastasis (15). According to epidemiological evidence, individuals with chronic inflammatory disorders have an increased risk of developing cancer. In addition, infections and inflammations were shown to be responsible for 15-20% of cancer-related deaths (16). Cancer cells are known to secrete cytokines, which are inflammatory chemicals that are implicated in the process of inflammation. Cytokines are crucial in sustaining chronic inflammation, promoting the growth of malignant epithelial cells, suppressing the anti-cancer response, and stimulating cancer proliferation (17). Studies have identified various cytokines and inflammatory elements that are linked to tumor formation, including IL-1, IL-4, and TNF (18, 19).

TME might impact the development of resistance to therapy (20). The options for affecting the TME are considered either direct or indirect immunotherapy. Direct immunotherapy strategies have an immediate impact on the cancer cells, whereas indirect immunotherapy strategies mostly target the surrounding microenvironment (21). These two therapeutic strategies are closely interconnected, with modifications in one resulting in alterations in the other. Immunotherapies specifically target cancer cells and disrupt the immunosuppressive microenvironment, which promotes anti-tumor immune responses and is currently gaining interest as an achievable and novel therapeutic option for cancers (22). Therefore, immunotherapy is a promising treatment approach for cancer, since it stimulates the immune system to identify and eliminate cancer cells.

As previously mentioned, immune-mediated therapies include a wide range of treatment approaches, such as oncolytic virotherapy, chimeric antigen receptor (CAR) T cells, and immunostimulatory cytokine (23). One of the most promising approaches is blocking immunological checkpoints (24). Recently, there has been significant attention on the programmed death receptor 1 (PD-1), which is one of the immune checkpoint inhibitors, that plays a role in suppressing T-cells and promoting self-tolerance (25). This will be discussed shortly in more detail.

### 2.1 Immunological checkpoint inhibitors

In the development of anticancer therapeutics, targeting immune checkpoint pathways has emerged as a highly promising strategy (26). Immune checkpoints are inhibitory regulatory mechanisms that maintain self-tolerance and modulate the magnitude and duration of immune responses in peripheral tissues (27). Key immune checkpoint molecules, including cytotoxic T lymphocyte antigen 4 (CTLA-4) and programmed cell death protein 1 (PD-1), are expressed on activated T cells and function to limit excessive immune activation. Tumor cells exploit these inhibitory pathways to evade immune surveillance, leading to suppression of T-cell function and the development of T-cell exhaustion (28, 29). Regulatory T cells (Tregs) further contribute to immunosuppression through high expression of CTLA-4 and PD-1, reinforcing inhibitory signaling within the tumor microenvironment (TME) (30, 31). In contrast, vascular endothelial growth factor (VEGF) signaling, mediated through VEGF receptors (VEGFR), plays a distinct role in tumor progression primarily by promoting angiogenesis (32). In addition to its pro-angiogenic function, VEGF can indirectly contribute to immunosuppression by enhancing Treg recruitment and impairing dendritic cell maturation. However, this mechanism is functionally and mechanistically distinct from classical immune checkpoint pathways (29).

Immune checkpoint inhibitors (ICIs) are now widely used across multiple cancer types, either as monotherapy or in combination with conventional treatments such as chemotherapy and radiotherapy (33, 34). Currently, the US FDA has approved three different types of ICIs, including PD-1 inhibitors (Nivolumab, Pembrolizumab, and Cemiplimab), PDL-1 inhibitors (Atezolimumab, Durvalumab, and Avelumab), and CTLA-4 inhibitor (Ipilimumab) that showed promising effectiveness in certain types of cancer, in particular, lung cancer and melanoma (35–37).

Ipilimumab, a monoclonal antibody (mAb) of the immunoglobulin 1 (IgG1) class that targets CTLA-4, was first approved as a therapeutic regimen for melanoma in 2010 (37). CTLA-4 is a member of the IgG class that inhibits the activation of T lymphocytes by binding to its ligands B7-1 (CD80) and B7-2 (CD86) expressed on antigen-presenting cells (APCs) (Sobhani et al., 2021a). Regarding cancer immunoregulation, CTLA-4 functions during the initial phase of T-cell activation in the lymph nodes, since its ligands are mostly expressed on APCs leading to inhibition of T-cell signaling (38). This indicates that blocking CTLA-4 can lead to the uncontrolled growth of T cells, which could enhance the immune response against tumors (39).

PD-1, also a member of the immunoglobulin superfamily, displays a significant coinhibitory function in the programmed cell death signaling triggered by the T-cell-mediated function (40). PD-1 is more widely expressed than CTLA-4 in several immune cell populations present in the TME, such as dendritic cells (DCs), natural killer (NK) cells, T cells, and B cells (41). Once PD-1 binds to its ligand PD-L1 (B7-H1), it initiates a signal that suppresses T-cell activity, leading to its eventual exhaustion (42). The understanding of the interaction between PD-1 and PD-L1 expressed in many tumor types, has concluded PD-L1 to be an interesting target for immunotherapy (43). Hence, specifically blocking the PD-1/PD-L1 signaling pathway has greatly improved the therapeutic options for various types of cancer, such as melanoma, head and neck squamous cell carcinoma, and non-small-cell lung cancer (NSCLC) (44). These advances have also paved the way for combination strategies integrating immune checkpoint inhibitors with oncolytic viruses, including adenovirus-based platforms, where viral-mediated tumor lysis and immune activation can synergize with checkpoint blockade to enhance anti-tumor responses.

### 2.2 Oncolytic viruses

Oncolytic viruses, known as OVs, are viruses that specifically target and eliminate cancer cells, while preferentially targeting cancer cells with minimal effects on normal tissues (45). Among these, oncolytic adenoviruses have been extensively engineered to enhance tumor selectivity and therapeutic efficacy (46). In addition to direct tumor cell lysis, OVs can be armed with immunostimulatory molecules to promote anti-tumor immune responses, making them key components of modern cancer immunotherapy (47, 48).

Currently, several oncolytic viruses (OVs) have received regulatory approval for cancer treatment following promising clinical outcomes (49) In 2005, the Chinese State Food and Drug Administration (SFDA) approved a genetically modified adenovirus, Oncorine (H101), developed by Shanghai Sunway Biotech, for the treatment of head and neck cancer. H101 is derived from human adenovirus serotype 5 and contains a deletion in the early region 1B 55-kDa (E1B-55K) gene, which encodes a protein known to interact with the tumor suppressor p53 (50). Early studies proposed that deletion of E1B-55K would confer tumor selectivity by restricting viral replication to p53-deficient cancer cells, as the E1B-55K protein was thought to be required to inhibit p53-mediated apoptosis in normal cells (51). However, subsequent research has demonstrated that this explanation is overly simplistic. Notably, *E1B-55K*–deleted adenoviruses, such as ONYX-015, were found to replicate in both *p53*-deficient and p53-proficient tumor cells, indicating that p53 status alone does not fully determine viral selectivity (52).

More recent evidence suggests that the E1B-55K protein plays a broader role in facilitating efficient viral replication, particularly through regulation of late viral mRNA export and modulation of host antiviral responses (53). The absence of *E1B-55K* can impair viral replication in normal cells due to inefficient viral mRNA transport and enhanced antiviral defense mechanisms, whereas many tumor cells exhibit defects in these pathways, allowing selective viral propagation (54). In addition to the *E1B* deletion, H101 also contains a partial deletion in the early region 3 (*E3*) gene, which may enhance its oncolytic properties (55). Therefore, tumor selectivity of *E1B-55K*–deleted adenoviruses is now understood to result from a combination of factors, including dysregulated cell cycle control, impaired antiviral responses, and altered RNA processing in cancer cells, rather than solely from *p53* deficiency (56). This revised understanding highlights the complexity of oncolytic adenovirus biology and underscores the importance of considering multiple tumor-specific pathways when designing effective OVs. Clinically, H101 is more effective when combined with Radio-chemotherapy in treating esophageal carcinoma, nasopharyngeal carcinoma, lung carcinoma, and hepatocellular carcinoma (55, 57, 58).

Moreover, in 2015, another OV originating from herpes simplex virus, type 1 (HSV-1) was engineered to express human granulocyte-macrophage colony-stimulating factor (GM-CSF) termed talimogene laherparepvec (T-VEC), was the first OV extensively approved in the USA and Europe for treating the recurrence of melanoma (59). The T-VEC was evaluated in many clinical trials in patients with advanced melanoma, and it showed an encouraging treatment response rate of 26%, which was significantly higher than when GM-CSF was administered alone (60–62). Therefore, oncolytic virotherapy is considered a promising approach to cancer immunotherapy.

Many studies have been exploring distinct types or subtypes of oncolytic virus (OVs) to assess their effectiveness and safety in treating diverse cancer types, including an extensive understanding of the virus’s biology and genomics (63). An efficient OV candidate must exhibit several distinctive features. For example, the OV should possess immunogenic properties, show oncolytic activity against cancer cells, and the limited pathogenicity in normal tissues (64). Furthermore, the OV must possess a high level of safety once administered in pre-clinical (48). Therefore, it is important to genetically engineer and arm with transgenic therapeutic agents to boost its ability to induce an anti-tumor immune response (65). Additionally, an OV must have the ability to modulate the processes of apoptosis and necrosis in target host cells, despite certain differences among different types of viruses (66, 67). Direct viral oncolysis represents a central mechanism underlying the therapeutic efficacy of oncolytic viruses. Lysis of infected tumor cells leads to the release of both pathogen-associated molecular patterns (PAMPs), derived from viral components, and damage-associated molecular patterns (DAMPs), originating from cellular debris (63). These signals are detected by pattern recognition receptors on innate immune cells, leading to activation of antigen-presenting cells, particularly dendritic cells, and induction of a pro-inflammatory tumor microenvironment (11).

This innate immune activation is accompanied by the release of pro-inflammatory cytokines, including type I interferons (IFN-α/β), tumor necrosis factor-alpha (TNF-α), and interleukin-12 (IL-12), which promote the recruitment and activation of natural killer (NK) cells and cytotoxic T lymphocytes (68). Importantly, tumor cell lysis also results in the release of tumor-associated antigens, which are processed and presented by dendritic cells to T cells, thereby initiating a robust adaptive anti-tumor immune response (69). However, this immune activation is often accompanied by upregulation of immune checkpoint molecules. For example, interferon signaling can induce the expression of PD-L1 on tumor cells and antigen-presenting cells, while activated T cells upregulate PD-1, leading to an immunosuppressive feedback loop that limits sustained anti-tumor activity (43). To overcome this limitation, oncolytic viruses are increasingly combined with immune checkpoint inhibitors or engineered to express checkpoint-blocking molecules as transgenes. These strategies include both combination approaches with systemic immune checkpoint inhibitors and the incorporation of checkpoint-blocking molecules as viral transgenes, each aiming to enhance T-cell activity while preserving the immunostimulatory effects of viral infection (64).

Genetic engineering of oncolytic viruses plays a critical role in improving tumor selectivity and therapeutic efficacy (70). This includes both deletion of viral genes required for replication in normal cells and insertion of immunostimulatory transgenes (71). For instance, in adenoviruses, deletion of E1A/E1B regions enhances tumor selectivity by restricting replication to cancer cells (72). Similarly, herpes simplex virus type 1 (HSV-1)-based oncolytic viruses are often engineered through deletion of neurovirulence genes, such as ICP34.5, to improve safety and tumor selectivity (73). Talimogene laherparepvec (T-VEC), a modified HSV-1, combines ICP34.5 gene deletion with insertion of the immunostimulatory transgene granulocyte-macrophage colony-stimulating factor (GM-CSF), thereby enhancing dendritic cell recruitment and antigen presentation (60, 74). Likewise, engineered HSV expressing interleukin-12 (IL-12) represents a transgene-based strategy that enhances anti-tumor immune responses, as demonstrated in early-phase clinical trials targeting glioblastoma (NCT02062827) (75).

Other viral platforms, including reovirus, rely primarily on natural tumor selectivity mechanisms, such as preferential replication in Ras-activated cells, although genetic modifications have also been explored to further enhance therapeutic efficacy (76, 77). Interestingly, many OVs are specifically engineered to target angiogenesis, whereas others are designed to eradicate tumor vasculature. Therefore, it is important for OVs to possess the ability to destroy blood vessels that nourish the tumor (78). For instance, studies have shown that modified HSV and vaccinia viruses can be engineered to exploit the pro-angiogenic tumor microenvironment, including elevated levels of fibroblast growth factor and vascular endothelial growth factor (VEGF), to enhance selective replication within tumor-associated vasculature (79, 80). Moreover, another study has revealed that the vesicular stomatitis virus (VSV) can potentially infect the blood vessels in the TME and induce thrombosis in the affected blood vessels (81). Collectively, these approaches highlight the importance of both viral engineering and immune modulation in optimizing oncolytic virotherapy.

### 2.3 Combining oncolytic virotherapy with immunotherapy strategies

Immunotherapy aims to stimulate the immune system to recognize and eliminate cancer cells through a variety of approaches, including immune checkpoint blockade, adoptive cell therapy, and oncolytic virotherapy. In this context, genetic engineering strategies, such as the insertion of transgenes encoding co-stimulatory molecules, cytokines, chemokines, or tumor-associated antigens into therapeutic vectors, represent one important approach to enhancing anti-tumor immune responses (82). In the past decade, with the development of recombinant gene therapy, it became possible to genetically modify DNA viruses to increase their affinity for tumor cells. This was first demonstrated in 1991 with herpes simplex virus type one (HSV-1) in an experimental glioma model (83). Oncolytic virotherapy can be utilized either as a vaccine to boost the immune system or for direct oncolysis triggered by virus replication (48), or by utilizing the potential of OVs in combination therapies with conventional cancer therapy strategies, such as ICIs and T cell-based treatments, which may increase the effectiveness of the anticancer response (84).

To enhance the effectiveness of OVs, researchers are developing different techniques to stimulate the TME and trigger the anti-tumor response that promotes the spread and immunogenicity of the OVs (64). In the process of generating a T cell-mediated response against cancer cells, the antiviral response can be activated to alter the TME from low immune activity (“cold”) to high immunological activity (“hot”) which enhances the infiltration of T-cells and cytokines production (85).

Despite the significant advances achieved with immunotherapeutic agents, which have demonstrated improved patient survival compared to conventional chemotherapy, their clinical application is now widely established across multiple malignancies and is no longer restricted to specific cancer types (48). However, the therapeutic efficacy of oncolytic viruses (OVs) as monotherapy remains relatively limited (86). Each viral vector system possesses a distinct set of characteristics that influence its suitability for cancer therapy, particularly in achieving efficient yet transient gene expression (87). To achieve optimal therapeutic outcomes, it is essential to deliver an appropriate dose of the therapeutic agent while minimizing toxicity to normal tissues (88).

In contrast, combination strategies integrating OVs with immune checkpoint inhibitors have demonstrated considerable therapeutic potential. Several studies have explored the efficacy of combining talimogene laherparepvec (T-VEC) with checkpoint inhibitors (82). In a phase I clinical trial, the combination of T-VEC and ipilimumab in patients with advanced melanoma resulted in objective responses in approximately 50% of patients, with durable progression-free survival (PFS) at 18 months and an overall survival (OS) rate of 67% (89). Furthermore, a phase II study reported an overall response rate of 39% for combination therapy, compared to 18% in patients treated with ipilimumab alone (90). These findings highlight the potential of OV-based combination strategies to overcome the limitations of monotherapy and enhance anti-tumor immune responses.

## 3 Principle of adenovirus-based vector for cancer therapy

Over the past few decades, adenoviral vectors have emerged as versatile platforms in cancer gene therapy and vaccinology, with more than 2,000 clinical trials conducted globally since 1989 (91) (92),. Adenoviruses (Ads) possess several advantageous properties, including broad cellular tropism, high transgene capacity, and the ability to infect both dividing and non-dividing cells (93). Importantly, adenovirus-based systems used in cancer therapy can be broadly divided into two distinct categories based on their replication capacity and therapeutic purpose: 1) Replication-defective (RD) adenoviral vectors, which are primarily used for gene therapy and vaccine delivery, and 2) Replication-competent oncolytic adenoviruses (OAds), which are specifically engineered to selectively replicate in and destroy cancer cells (13, 94, 95). These platforms are mechanistically and functionally distinct and should not be considered interchangeable. RD vectors are designed for safe transgene delivery without viral replication, whereas OAds retain or modify replication machinery to enable tumor-selective oncolysis and immune activation (96).

### 3.1 Molecular biology and adenovirus genome

**Figure 1:**
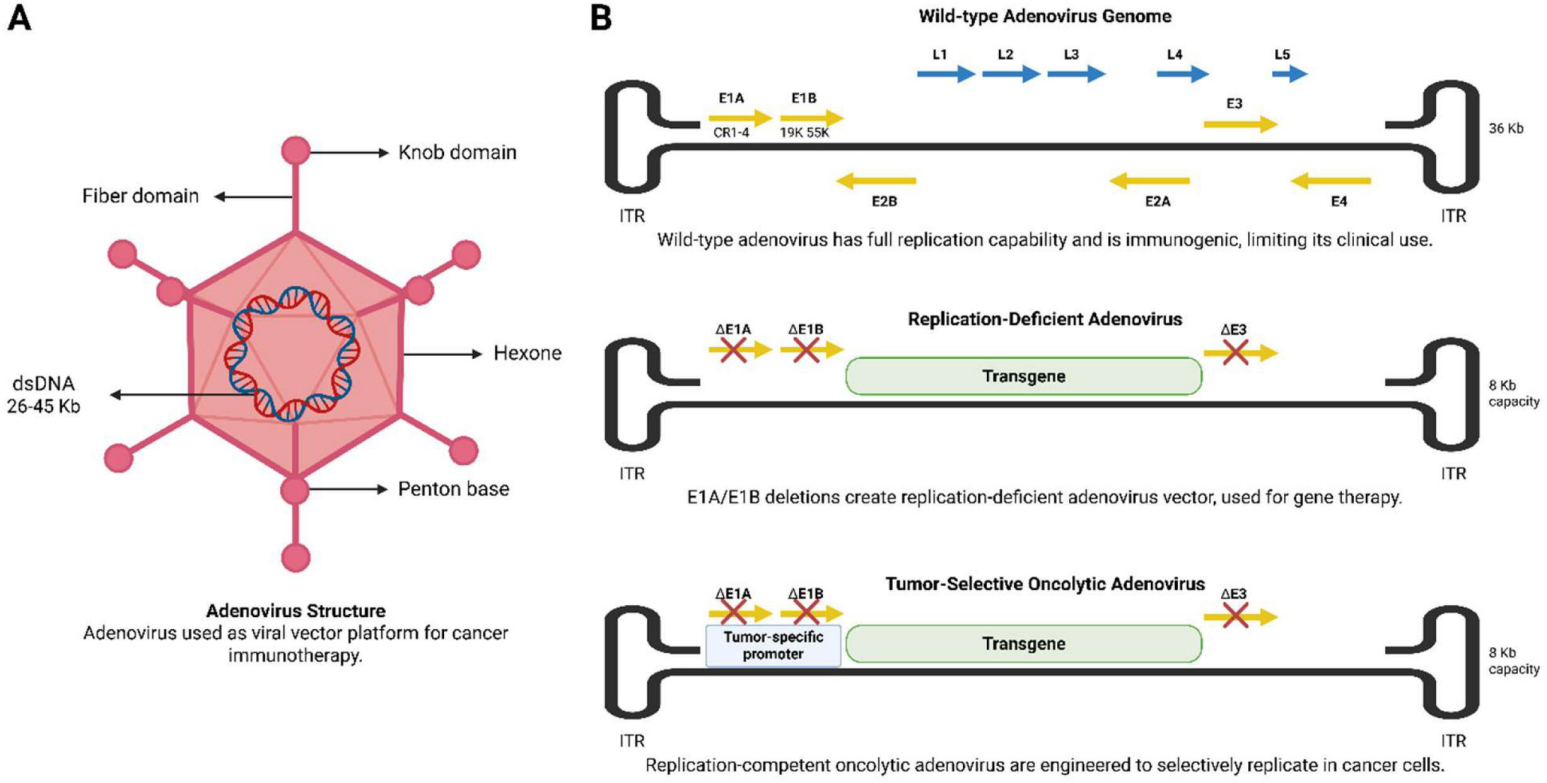
Schematic representation of adenovirus structure and genome organization, along with major engineering strategies used for therapeutic applications. **(A)** Schematic representation of adenovirus structure, highlighting key capsid components including the knob domain, fiber domain, hexon, and penton base. The viral genome consists of double-stranded DNA (26–45 kb), which can be modified for therapeutic use. **(B)** Comparison of adenoviral genome modifications. The wild-type adenovirus contains intact E1A, E1B, E3, and E4 regions and retains full replication capacity but is limited by host immunogenicity. In replication-deficient adenoviruses, deletions in E1A and E1B prevent viral replication, allowing for safe delivery of therapeutic transgenes. Tumor-selective oncolytic adenoviruses retain the ability to replicate, but only in tumor cells, via insertion of E1A under a tumor-specific promoter. Deletion of E3 further increases space for transgene insertion and modulates host immune responses.

Adenoviruses are non-enveloped double-stranded DNA viruses, consisting of an icosahedral capsid made up of 240 hexagons forming the faces and structural basis of the viral capsid structure which contains the core protein and linear viral DNA genome around 26–45 kb (97, 98). At the vertices, the icosahedral capsid has 12 pentons and slender fibers formed out of the fivefold position of the shell, which is involved in the attachment and entry of the Ads into host cells (**Figure 1A**) (99).

Adenoviruses derived their name from the first isolated polioviruses in human adenoid cell culture in 1953 by Rowe and colleagues (100). Ads have been isolated from several species, such as primates, bovines, fowls, reptiles, and humans. Human Ads (HAdVs) are categorized into more than 60 immunological serotypes, divided into 6 subgroups, which include seven known HAdV species HAdV-A to HAdV-G (101, 102). Among HAdV, only some serotypes cause severe infections that mainly infect respiratory and gastrointestinal epithelia, which leads to meningitis, conjunctivitis, gastroenteritis, and acute hemorrhagic cystitis (103, 104). Most of these infections primarily affect children under the age of 5 (105), and immunocompromised individuals (106, 107).

Studies have significantly advanced the understanding of adenovirus (Ad) attachment and entry by highlighting the diversity of viral–host receptor interactions, including coxsackievirus and adenovirus receptor (CAR) and CD46 (108). In addition to these well-characterized receptors, different adenovirus species utilize a broader range of cellular entry molecules, contributing to their distinct tissue tropism. For example, species B adenoviruses, such as HAdV-3 and HAdV-35, primarily use CD46, whereas species D adenoviruses have been reported to interact with sialic acid-containing glycans (108). Furthermore, certain serotypes can engage desmoglein-2 (DSG-2), a junctional adhesion molecule, to facilitate viral entry (109). This diversity in receptor usage provides a foundation for the development of chimeric adenoviral vectors with altered tropism and improved targeting efficiency (110). Such insights have important implications for both drug design and gene therapy applications, enabling the rational engineering of adenoviral vectors for therapeutic purposes.

While CAR remains the primary receptor for species C adenoviruses, including serotypes 2 and 5 (111, 112) other receptor interactions remain less well characterized. Upon attachment to the host cell, the fiber protein dissociates, exposing the penton base (113). The penton base contains an Arg–Gly–Asp (RGD) motif that binds to integrins such as αvβ3 and αvβ5, facilitating viral internalization via receptor-mediated endocytosis (108, 114). Following internalization, the viral capsid undergoes disassembly, allowing the viral genome to be transported into the host nucleus (115). Collectively, these receptor interactions contribute to the broad tropism of adenoviruses and highlight the importance of receptor diversity in determining infection efficiency and therapeutic targeting (116, 117). Considering the intrinsic infectivity of adenoviruses and their ability to utilize diverse endocytic pathways for cellular entry, they have emerged as highly versatile platforms for gene therapy and therapeutic delivery, particularly in cancer applications. Recent advances in adenoviral vector engineering and clinical translation have further reinforced their potential in modern molecular medicine (118).

The complete genome of several human adenovirus serotypes has been fully sequenced, including Ad2 and Ad5, which have approximately 36 kb and encode about 40 proteins (119). The protein-coding regions are classified as early or late based on their expression either before or after DNA replication (97). The early genes E1A, E1B, E2, E3, and E4 are transcribed first and coding genes responsible for triggering the transcription of other viral structural genes and regulating the host cellular environment to facilitate viral replication, while the late genes (L1–L5) mostly encode the viral structural proteins (120). Adenovirus also displays various proteins, including E1B55K, E4orf3, E4orf4, E4orf6, and core protein VII, that suppress the host’s DNA damage response (DDR), which might otherwise inhibit adenoviral reproduction (97, 99).

The early region 1A (E1A) of human adenovirus types 2 and 5, expressed during adenovirus replication, encodes two major proteins, 12S and 13S, which are essential for initiating viral replication (121). The E1A 13S protein consists of 289 amino acids and contains four conserved regions (CR1–CR4) (122). In infected cells, the CR2 region of the HAdV E1A protein binds retinoblastoma (RB) family proteins, including RB and RB-related proteins (123). This interaction releases E2F transcription factors from RB-mediated repression, leading to de-repression of E2F and subsequent entry of the cell into the S-phase (121). This cell cycle modulation is crucial for creating a cellular environment that supports efficient viral replication (124).

The HAdV E1B gene encodes two major proteins: E1B-55 kD and E1B-19 kD (125). The E1B55K protein binds to the tumor suppressor p53 to inhibit the initiation of apoptosis induced by E1A in infected cells (47). The HAdV E1B19K protein functions as a potent inhibitor of apoptosis by disrupting the functions of the pro-apoptotic proteins (111). The E1b-encoded proteins together prevent premature cell death and augment viral replication (126).

E2 proteins play vital roles during viral DNA replication, whereas E3 and E4 proteins affect host immunological responses and cell signaling (127). In particular, E3 proteins suppress antiviral immune responses by affecting antigen presentation cells, cytokines, and apoptotic pathways, although they are not necessary for viral replication (128). Furthermore, E4 encoded genes exhibits regulatory activities that promote late gene transcription and translation for required viral replication (127). Late genes encode structural capsids of HAdV, such as penton, hexon, fiber, and core viral proteins (129). Adenoviral vectors are frequently genetically modified, such as through deletions in the E1A, E1B, or E3 regions, to enhance safety, increase transgene capacity, or enable tumor-selective replication. The functional implications of these modifications differ depending on whether the vector is replication-defective or oncolytic, as discussed in subsequent sections (**Figure 1B**) (130, 131).

### 3.2 Antiviral Immune Responses to Adenoviruses

Human adenoviruses (HAdVs) elicit robust host immune responses upon infection. They activate multiple innate immune signaling pathways, leading to the production of pro-inflammatory cytokines that subsequently drive strong humoral and cellular adaptive immune responses (132). production of pro-inflammatory cytokines that subsequently drive strong humoral and cellular adaptive immune responses (133). These immune responses are primarily directed against viral structural proteins, including hexon and fiber components, which serve as major antigenic targets (134, 135).

Both *in vitro* and *in vivo* studies have demonstrated that HAdV infection induces the production of type I interferons (IFN-I), particularly by plasmacytoid dendritic cells (pDC), which play a critical role in orchestrating both innate and adaptive immunity (136). Furthermore, another study explained that the suppression of IFN-I in mice reduced the adaptive and the innate immune responses to HAdV, resulting in more sustained transgene expression and decreased inflammatory responses *in vivo* (137). These findings highlight the importance of understanding innate immune activation mechanisms to optimize the safety and efficacy of adenoviral vectors in gene therapy and vaccine development.

Replication-deficient adenovirus vectors are widely used as vaccines due to their ability to induce robust humoral and T-cell responses against encoded transgenes (138). In the context of oncolytic adenoviruses, the host immune response plays a dual role. While antiviral immunity may limit viral persistence and spread, it can also enhance anti-tumor efficacy (139). Viral infection of tumor cells leads to the presentation of viral antigens on the surface of infected cells, which can promote immune recognition and cytotoxic killing, even in cases where tumor-associated antigens are weakly immunogenic (140).

In addition, pre-existing cellular immunity to adenoviruses can be reactivated and redirected toward the tumor site, contributing to the elimination of infected cancer cells (141). Although reducing early immune clearance may be beneficial, particularly for systemic administration where neutralizing antibodies can limit viral delivery, the immunogenicity of oncolytic adenoviruses is not inherently detrimental (142). In contrast, in intratumoral delivery settings, localized immune activation can enhance both antiviral and anti-tumor responses (49, 93). Therefore, rather than completely suppressing antiviral immunity, current strategies aim to balance viral persistence with immune activation, often through combination approaches involving immunomodulatory agents or therapeutic transgenes to optimize anti-tumor efficacy.

## 4 Oncolytic adenoviruses construction

Adenoviruses have been extensively engineered for therapeutic applications due to their well-characterized genome, high transgene capacity, and ability to induce robust immune responses (143). Importantly, adenoviral platforms encompass distinct applications, including gene therapy vectors, vaccine vectors, and oncolytic adenoviruses, which differ fundamentally in their design and therapeutic objectives. While adenoviral vectors are widely used in gene therapy and vaccine development, it is important to clearly distinguish these applications from oncolytic adenovirus strategies. HAdVs represent one of the most extensively studied and widely utilized viral vectors in clinical applications, owing to their well-characterized genome, high transgene capacity, and ability to infect both dividing and quiescent cells (119).

Replication-defective adenoviral vectors are generated through deletion of essential viral genes, most commonly E1A and E1B, rendering them incapable of replication. These vectors are primarily used for transgene delivery, including therapeutic gene replacement and vaccination, where controlled expression rather than viral propagation is desired (127, 144). In contrast, oncolytic adenoviruses (OAds) are designed to retain replication capability but in a tumor-selective manner. These vectors exploit cancer-specific molecular defects or regulatory elements to enable selective viral replication within tumor cells while sparing normal tissues (145).

Thus, three different generations of adenoviral vectors have been constructed to enhance the ability to carry genes and improve safety (146). However, Adenoviral genomes have undergone progressive engineering to enhance tumor selectivity and therapeutic function. These modifications range from early-generation replication-deficient vectors to advanced tumor-selective and transgene-armed constructs. In the first generation, E1 and E3 genes were deleted to produce the replication-defective adenoviral vector (147). However, the deletion of viral genes creates around 8 kb allowing the vector to efficiently deliver transgenes into host cells without leading to cell death (148). The main limitation of first-generation adenoviral vectors is the notable cellular toxicity, causing inflammation and induce strong immune response (149).

In second-generation adenoviral vectors, the E2 and E4 regions are deleted in addition to the E1/E3 genes deletion (150). The second-generation adenoviral vectors offer increased capacity for larger sequences up to 10.5 kb (151). Compared to the first generation, the second generation’s titers were significantly lower, but the immunogenicity and cellular toxicity remain a significant issue (150, 152). Third-generation adenoviral vectors, also known as high-capacity adenoviral vectors (HCAds), can carry cargo sequences up to 36 Kb (153). The HCAds were created by deleting all viral genes retaining only necessary cis-acting elements of Inverted Terminal Repeats (ITRs) and packaging, and for viral replication, thus, an adenoviral helper virus is required instead of a complemented cell line (154, 155).

Third-generation vectors offer many advantages compared to first and second-generation vectors, including increasing the exogenous capacity up to 36 kb, offering less cellular toxicity while safety being significantly enhanced (156). On the other hand, HCAds are more complex compared to first and second-generation adenoviral vectors and their purification process requires the removal of helper viruses which has the possibility of helper virus contamination (157). This leads to an increase in complexity and costs, thus posing challenges for clinical application (158).

Adenovirus vectors are mostly classified into two categories replication-defective and replication-competent (oncolytic) vectors (159). Replication-defective vectors are primarily utilized for transient gene expression in gene therapy and vaccination settings, where controlled transgene delivery is required without viral replication (138). While these generations are critical for gene therapy and vaccine applications, oncolytic adenoviruses follow a different design logic, where selective replication—rather than complete attenuation—is the primary goal (160). These fundamental differences in design logic underscore that replication-defective vectors, vaccine vectors, and oncolytic adenoviruses are not interchangeable platforms but rather serve distinct therapeutic purposes.

The Replication-defective (RD) vectors lack the E1 genes essential for viral replication and are replaced by an expression of exogenous cassette controlled by a highly effective promoter which is responsible for regulating the expression of the transgene cassette, such as the cytomegalovirus (CMV) immediate early promoter (161). Thereby, the deletion of E1 is often used to generate replication-defective adenoviral vectors used as shuttle vectors in gene therapy (132, 162). Due to an increase in the viral capacity, non-essential sections of viral genes, such as the E3 region, are often deleted to increase the capacity to introduce a therapeutic gene into a recombinant adenoviral genome (163). Replication-defective adenoviruses cannot be replicated in the absence of *E1* complementation and therefore require packaging cell lines such as HEK293 (164).

**Figure 2:**
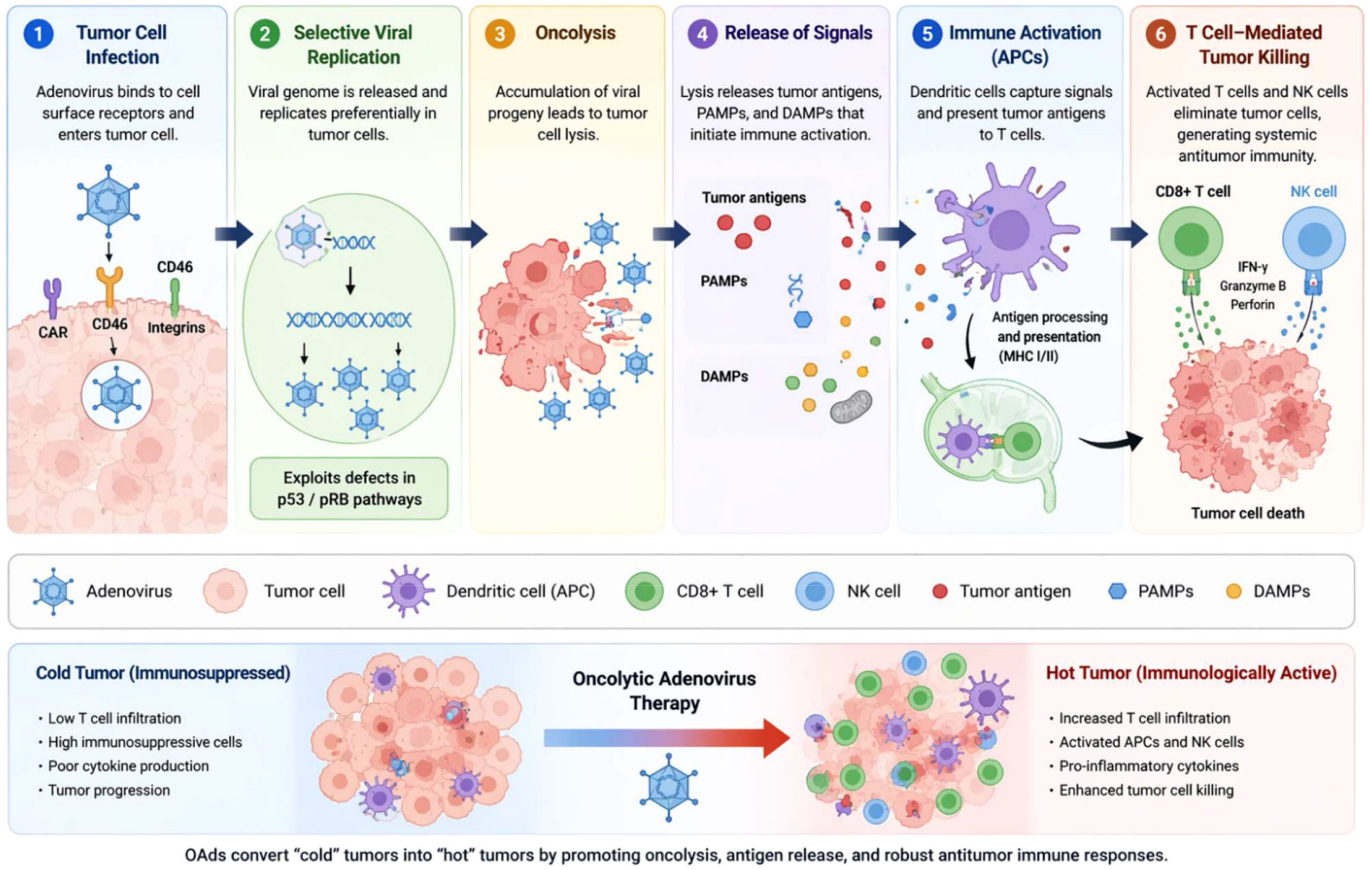
Mechanisms of antitumor activity of oncolytic adenoviruses. Schematic overview of the mechanisms underlying oncolytic adenovirus (OAd)–mediated antitumor activity. OAds selectively infect and replicate within tumor cells, leading to direct oncolysis and release of tumor-associated antigens, pathogen-associated molecular patterns (PAMPs), and damage-associated molecular patterns (DAMPs). These signals promote dendritic cell activation and antigen presentation, initiating adaptive immune responses characterized by cytotoxic T-cell activation.

In contrast to RD vectors, OAds are engineered to achieve conditional replication in tumor cells through multiple strategies, one major strategy involves modifying early viral genes such as E1A and E1B to achieve tumor-selective replication, as discussed in detail in Section 4.1.1 (165). Unlike RD vectors, OAds maintain the ability to undergo a lytic replication cycle, leading to direct tumor cell destruction (oncolysis). This process is accompanied by the release of tumor-associated antigens, as well as pathogen-associated molecular patterns (PAMPs) and damage-associated molecular patterns (DAMPs), which collectively stimulate innate and adaptive immune responses. As illustrated in **Figure 2**, viral infection of tumor cells initiates a cascade of immunological events, including dendritic cell activation, antigen presentation, and subsequent priming of cytotoxic T lymphocytes. These immune mechanisms contribute to systemic antitumor effects and support the conversion of immunologically “cold” tumors into “hot” tumors (160).

The first oncolytic adenovirus, ONYX-015 (dl1520), was engineered with a deletion in the E1B55K region and evaluated in clinical trials for the treatment of head and neck cancer (165). Although initial results demonstrated safety in intratumoral administration, its therapeutic efficacy in metastatic settings was limited, and clinical development was eventually discontinued (166, 167). A similar strategy was later employed in the development of H101 (Oncorine), which also contains an E1B55K deletion. In 2005, H101 was approved by the Chinese State Food and Drug Administration (SFDA) for the treatment of head and neck cancer, particularly in combination with chemotherapy (50, 168).

### 4.1 Strategies for enhancing tumor selectivity and therapeutic efficacy

To improve the therapeutic performance of oncolytic adenoviruses, multiple complementary strategies have been developed, including genetic targeting of viral replication, capsid modification to enhance tumor tropism, and arming with therapeutic transgenes to stimulate antitumor immunity.

Early clinical observations of adenovirus-mediated tumor regression date back to the 1950s; however, safety and efficacy limitations restricted their development (169). Notably, approximately 65% of patients exhibited tumor regression without major toxicity, highlighting the early therapeutic potential of adenoviral approaches (169). Representative clinical trials evaluating adenoviral vectors for cancer immunotherapy are summarized in **Table 1**, clinical trial information is based on data available from ClinicalTrials.gov.

**Table 1:** Completed and ongoing clinical trials using adenoviral vectors for cancer immunotherapy.

Adenovirus	Backbone	Tumor target	Transgene (s)	NCT number	Phase
ONCOS-102	Ad5/3 chimeric, Δ24	Malignant solid tumors	GM-CSF	NCT01598129	Phase I
LOAd703	Ad5/35 chimeric	Pancreatic, melanoma, colorectal cancer	CD40L, 4-1BBL	NCT03225989; NCT04123470; NCT02705196	Phase I–II
Ad5-yCD/mutTKSR39rep-hIL-12	Replication-competent Ad5	Prostate cancer	yCD, TK, IL-12	NCT02555397	Phase I
ADV/HSV-tk	Oncolytic Ad5	NSCLC, TNBC	HSV-tk	NCT03004183	Phase II
TBX-071/ETBX-061/ETBX-051	Ad5 ΔE1, ΔE2B	Prostate cancer	PSA, MUC1, brachyury	NCT03481816	Phase I
OBP-301 (Telomelysin)	Ad5	Esophagogastric adenocarcinoma	—	NCT03921021	Phase II
Ad/HER2/Neu DC	Ad5-based (Ad35 fiber knob)	Breast cancer	HER2 (ECTM domain)	NCT01730118	Phase I
Ad/PNP	Ad5 ΔE1–E3	Head and neck cancer	E. coli PNP	NCT01310179; NCT03754933	Phase I–II
Ad-RTS-hIL-12	Ad5 ΔE1–E3	Glioblastoma	Regulated IL-12 (RTS system)	NCT02026271	Phase I
Ad.p53-DC vaccine	Ad5	Small cell lung cancer	p53	NCT00617409	Phase II
Adenovirus/PSA	Ad5	Prostate cancer	PSA	NCT00583752	Phase II
DNX-2401	Ad5 Δ24	Glioblastoma	RGD-4C motif	NCT00805376; NCT02197169; NCT02798406	Phase I–II
AdCD40L	Ad5 ΔE1–E3	Melanoma	CD40L	NCT01455259	Phase I–II
CELYVIR	Ad5 ΔE1A/E1B	Metastatic tumors	MSC-delivered Ad	NCT01844661	Phase I–II

GM-CSF, granulocyte-macrophage colony-stimulating factor; IL-12, interleukin-12; PSA, prostate-specific antigen; MSCs, mesenchymal stem cells.

**Figure 3:**
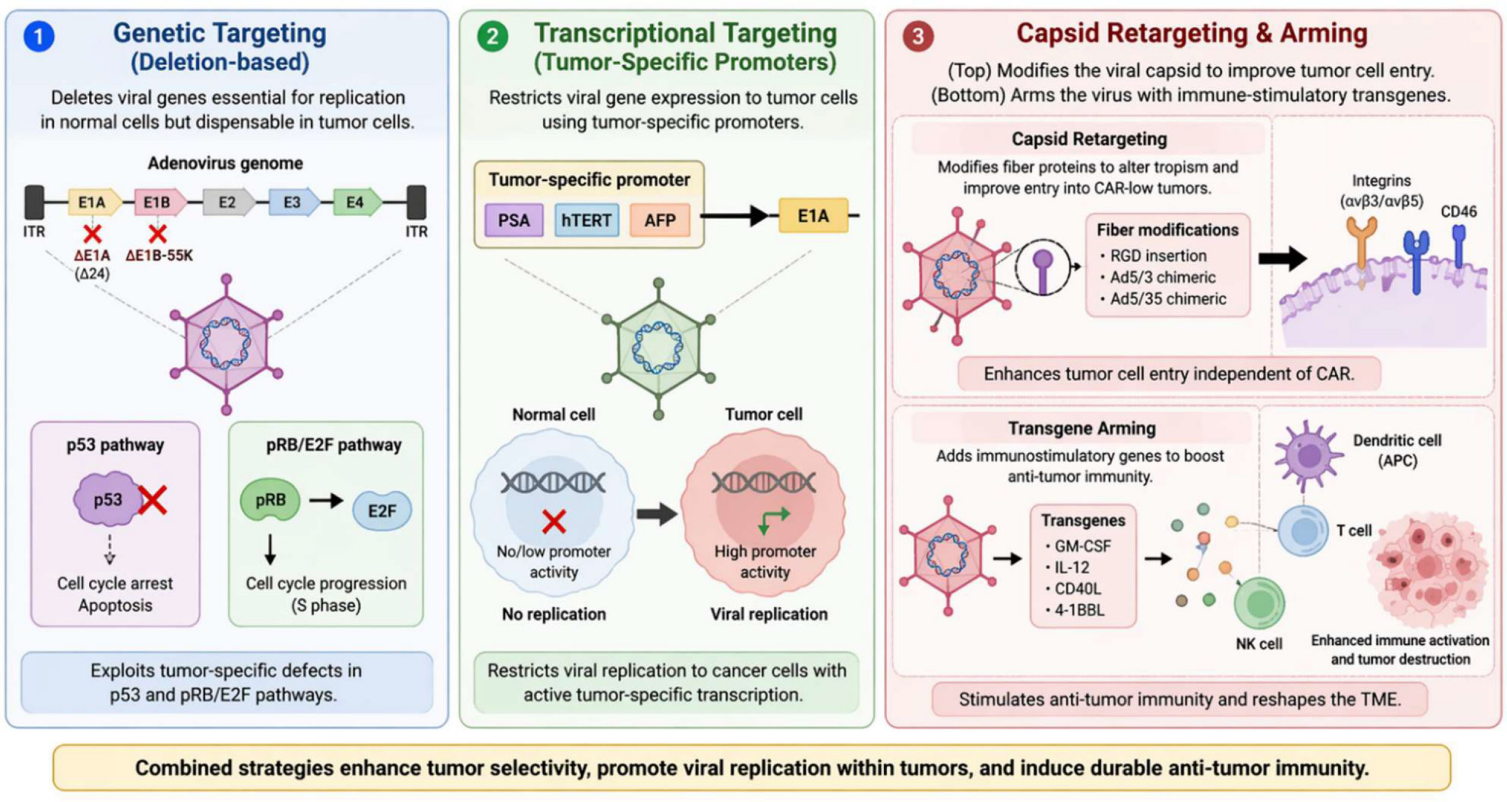
Classification of tumor-selective targeting strategies in oncolytic adenoviruses. Oncolytic adenoviruses (OAds) are engineered using multiple complementary strategies to enhance tumor selectivity and therapeutic efficacy. (1) Genetic targeting involves deletion of viral genes such as *E1A* and *E1B* to exploit tumor-specific defects in *p53* and *pRB*/EF pathways. (2) Transcriptional targeting utilizes tumor-specific promoters (e.g., PSA, hTERT, AFP) to restrict viral replication in cancer cells. (3) Capsid retargeting modifies viral surface proteins (e.g., RGD insertion, Ad5/3, Ad5/35 chimerism) to improve infection of tumor cells with low CAR expression. In addition, transgene arming enables expression of immunostimulatory molecules such as GM-CSF, IL-12, CD40L, and 4-1BBL, enhancing antitumor immune responses.

Despite these promising findings, further optimization is required to enhance tumor selectivity and therapeutic efficacy. Current strategies focus on engineering oncolytic human adenoviruses (HAdVs) through modifications such as partial deletions of essential viral genes and the use of tumor-specific promoters to achieve selective replication in cancer cells (65, 170). More broadly, modern oncolytic adenovirus design strategies can be categorized into genetic targeting of viral replication, capsid modification to alter viral tropism, and transgene arming to enhance immune-mediated antitumor activity as shown in **Figure 3** (69, 71).

#### 4.1.1 Genetic Targeting Strategies

As mentioned previously, human adenoviruses (HAdVs) exhibit promising oncolytic properties but require genetic modifications to achieve tumor-selective replication (171). These modifications exploit molecular differences between normal and cancer cells, particularly defects in cell cycle regulation and antiviral responses. One major approach involves deletion-based targeting, in which viral genes essential for replication in normal cells are removed but remain dispensable in tumor cells. A well-known example is the deletion of the *E1B55K* gene, as seen in ONYX-015 (dl1520). Although initially proposed to confer selectivity for *p53*-deficient tumors, subsequent studies have demonstrated that this mechanism is more complex and not solely dependent on *p53* status (172, 173).

A second major approach targets the retinoblastoma (*pRB*) pathway. In normal cells, *pRB* regulates cell cycle progression by sequestering E2F transcription factors, thereby preventing entry into the S-phase (174). In many cancer cells, this regulatory pathway is disrupted (175). The adenoviral *E1A* protein normally binds *pRB*, releasing E2F and promoting viral replication (51). Deletions within the *E1A-CR2* region, such as the Δ*24* mutation, prevent this interaction in normal cells while allowing selective replication in tumor cells with defective *pRB* signaling (176–178).

In addition to deletion-based strategies, transcriptional targeting has been widely employed (179). This approach utilizes tumor-specific promoters to control the expression of essential viral genes such as E1A (180). These promoters exploit aberrant transcriptional activity in cancer cells, thereby restricting viral replication to tumor tissue (135, 181). Examples include prostate-specific antigen (PSA), human telomerase reverse transcriptase (hTERT), and alpha-fetoprotein (AFP) promoters (172, 182, 183). Several engineered OAds, including CN706, ICOVIR variants, and SynOV1.1, have demonstrated improved selectivity using this strategy (96, 184–186).

#### 4.1.2 Capsid modification and tumor tropism

In addition to controlling viral replication, enhancing tumor selectivity can be achieved by modifying viral tropism (187). Adenovirus type 5 (HAdV5), the most used backbone, relies on the coxsackievirus and adenovirus receptor (CAR) for cell entry. However, CAR expression is often reduced in many tumor types, limiting infection efficiency (188).

To overcome this limitation, capsid engineering strategies have been developed to retarget adenoviruses toward alternative cellular receptors (189). One approach involves the use of chimeric fiber proteins, such as Ad5/3 and Ad5/35, which replace the native fiber knob with that of other adenovirus serotypes to enhance tumor cell infectivity (190). The chimeric 5/35 adenoviral vector (HAdV5/35) showed higher cell infection of gastric (185), and hepatic cancer cells (191). In addition, the chimeric viruses HAdV5/37 and HAdV3/11p have been developed and evaluated in clinical trials to enhance the transduction of transfer transgenes (192, 193).

Another strategy involves incorporating arginine–glycine–aspartic acid (RGD) motifs into the fiber knob or hexon proteins, enabling viral binding to integrins that are frequently overexpressed in tumor cells (176). For instance, melanoma cells lacking CAR from metastases of patients, have exhibited resistance to wild-type HAdV infection and cell apoptosis (194). Enhanced infection of cells with low CAR expression has been achieved by incorporating the RGD motif into the HI loop of the fiber knob domain or into the hexon protein of the HAdV that facilitates binding to the host cell through integrins (195, 196). Similarly, polylysine (pk7) modifications facilitate binding to heparan sulfate proteoglycans, further improving tumor cell targeting (70). These capsid modifications significantly enhance viral entry into tumor cells, particularly those with low CAR expression, thereby improving the overall effectiveness of oncolytic adenovirus therapy (197).

#### 4.1.3 Arming strategies to enhance antitumor response

Beyond direct tumor lysis, oncolytic adenoviruses can be further engineered to express therapeutic transgenes that enhance antitumor immune responses (71). This strategy, often referred to as “arming,” has emerged as a key advancement in oncolytic virotherapy (198). Armed OAds can express immunostimulatory cytokines such as granulocyte-macrophage colony-stimulating factor (GM-CSF), interleukin-12 (IL-12), and interferons, which promote immune cell recruitment and activation within the tumor microenvironment (199). For example, CG0070 and ONCOS-102 are GM-CSF–expressing adenoviruses that have demonstrated promising results in clinical and preclinical studies (200).

In addition, OAds expressing co-stimulatory molecules such as CD40L and 4-1BBL have shown enhanced activation of T-cell–mediated immune responses (201). The oncolytic adenovirus LOAd703, which expresses both CD40L and 4-1BBL, has demonstrated efficacy in targeting pancreatic cancer (202, 203). Combination strategies have also been explored, particularly the integration of OAds with immune checkpoint inhibitors (204). For instance, DNX-2401 combined with anti-PD-1 therapy has shown enhanced antitumor activity in glioma models, highlighting the synergistic potential of these approaches (199, 201). To provide a structured overview of these tumor-selective and therapeutic strategies, key approaches are summarized in **Table 2**. Overall, arming strategies extend the therapeutic function of OAds beyond direct oncolysis by reshaping the tumor microenvironment and promoting durable antitumor immunity (202).

**Table 2:** Tumor-selective design strategies in oncolytic adenoviruses.

Strategy	Mechanism	Genetic modification	Example viruses	Targeted pathway/feature
Deletion-based targeting	Removes viral genes required for replication in normal cells but dispensable in tumor cells	ΔE1B-55K; ΔE1A (Δ24)	ONYX-015, H101, DNX-2401	p53 dysfunction; pRB/E2F dysregulation
Transcriptional targeting	Restricts viral gene expression using tumor-specific promoters	E1A under tumor-specific promoters (e.g., hTERT, PSA, AFP)	CN706, ICOVIR-5, ICOVIR-7, VCN-01, SynOV1.1	Tumor-specific transcriptional activity
Capsid retargeting	Modifies viral tropism to enhance infection of tumor cells with low CAR expression	Fiber knob substitution (Ad5/3, Ad5/35); RGD insertion; pk7 modification	Ad5/3, Ad5/35, CRAd-Survivin-pk7	CAR-independent entry; integrins; CD46; heparan sulfate proteoglycans
Arming strategies	Enhances antitumor efficacy by expressing therapeutic transgenes	Insertion of immune-stimulatory genes (e.g., GM-CSF, IL-12, CD40L, 4-1BBL)	CG0070, ONCOS-102, LOAd703	Immune activation; TME modulation

CAR, coxsackievirus and adenovirus receptor; GM-CSF, granulocyte-macrophage colony-stimulating factor; IL-12, interleukin-12; TME, tumor microenvironment.

## 5 Discussion

In recent decades, adenovirus-based cancer vaccines and oncolytic viruses have evolved from initial preliminary investigations to innovative genetically modified platforms currently in clinical evaluation. Adenoviral vectors are distinguished by their well-defined genome, substantial transgenic capacity, and robust immunogenicity, making them notable prospects in cancer immunotherapy. However, despite significant preclinical achievements and promising results in certain clinical trials, the broad clinical application of recombinant adenoviruses is hindered by various biological and translational limitations.

The most significant advantage of oncolytic adenoviruses is their dual method of action: direct lysis of cancer cells and indirect stimulation of systemic anti-tumor immunity. Viral replication in cancer cells triggers immunogenic cell death, releasing tumor-associated antigens and signals that enhance immune cells activation. The ability to alter the tumor microenvironment from an immunologically “cold” to a “hot” status provide a strong rationale for using adenoviral platforms into cancer vaccination approaches. Nonetheless, the intensity and effectiveness of these immune responses differ significantly among tumor types, demonstrating the significance of tumor-intrinsic variables such as receptor expression, antiviral signaling capability, and baseline immune infiltration.

Genetic engineering techniques have significantly enhanced the tumor selectivity and safety characteristics of adenoviral vectors. Strategies such as E1A and E1B modifications, tumor-specific promoter regulation, and capsid retargeting have facilitated conditional replication mostly in malignant cells. Although these techniques minimize off-target toxicity, they may also impair viral viability and replication efficiency, thereby limiting therapeutic efficacy. This trade-off highlights a continual challenge in adenoviral design: achieving an ideal balance between safety and oncolytic effectiveness. Future vector development may gain from combinatorial designs that incorporate numerous layers of selectivity while maintaining significant replicative capacity.

A significant limitation is the host’s antiviral immunological response. Pre-existing immunity to prevalent adenovirus serotypes, especially Ad5, can significantly affect vector delivery efficacy and transgenic expression after systemic administration. This immunogenicity, while beneficial for vaccine applications, provides a challenge for repeated doses and prolonged therapeutic effects in cancer treatment. A comprehensive understanding of how innate immune signaling pathways affect therapeutic and antiviral immunity is crucial for the rational optimization of vectors.

The clinical outcomes using adenovirus-based monotherapies have typically been modest, suggesting that oncolytic adenoviruses are unlikely to be effective as independent therapies for a majority of solid tumors. On the other hand, combined approaches— particularly that involving immune checkpoint inhibitors—have exhibited synergistic effects by enhancing T-cell infiltration and eliminating adaptive immunological resistance mechanisms.

In conclusion, recombinant adenoviruses provide an efficient and potent platform for cancer vaccines and immunotherapy. Despite significant advancements in vector design and combinations strategies, considerable development is required for overcoming immune-related limitations and improve therapeutic efficacy. Such efforts will be essential to fully realize the potential of adenovirus-based cancer vaccines in cancer immunotherapy.
